# Application and Prospect of a Mobile Hospital in Disaster Response

**DOI:** 10.1017/dmp.2020.113

**Published:** 2020-04-22

**Authors:** Xinlin Chen, Lu Lu, Jie Shi, Xin Zhang, Haojun Fan, Bin Fan, Bo Qu, Qi Lv, Shike Hou

**Affiliations:** Institute of Disaster Medicine, Tianjin University; Tianjin Key Laboratory for Disaster Medicine Technology, Tianjin, China

**Keywords:** application, disaster medicine, disaster response, mobile hospital

## Abstract

Disasters such as an earthquake, a flood, and an epidemic usually lead to large numbers of casualties accompanied by disruption of the functioning of local medical institutions. A rapid response of medical assistance and support is required. Mobile hospitals have been deployed by national and international organizations at disaster situations in the past decades, which play an important role in saving casualties and alleviating the shortage of medical resources. In this paper, we briefly introduce the types and characteristics of mobile hospitals used by medical teams in disaster rescue, including the aspects of structural form, organizational form, and mobile transportation. We also review the practices of mobile hospitals in disaster response and summarize the problems and needs of mobile hospitals in disaster rescue. Finally, we propose the development direction of mobile hospitals, especially on the development of intelligence, rapid deployment capabilities, and modularization, which provide suggestions for further research and development of mobile hospitals in the future.

Natural and man-made disasters have become world-wide global concerns.^1^ Many types of disasters, such as earthquakes, tsunamis, hurricanes, floods, explosions, epidemics – severe acute respiratory syndrome (SARS), Ebola, coronavirus disease 2019 (COVID-19), influenza – and terrorist attacks, seem to be growing in number and intensity. Medical response in time to disaster is key for the health care of mass casualties.^2^ After the disaster, in the face of the destruction and paralysis of the local medical institutions, it is very important to quickly and effectively deploy the external medical rescue platform centered on the mobile hospital. A mobile hospital is a kind of hospital that takes on the early treatment of patients and some specialized treatment with the aid of mobile transportation capacity. It can be composed of a medical unit, a technical support unit, a ward unit, a life support unit, and so forth. A mobile hospital has the advantages of good mobility, strong adaptability to the environment, rapid deployment, complete medical functions, early treatment of the wounded, and so forth, so it is widely valued by all countries. Therefore, in the face of the disaster rescue with a complex natural environment and a large number of casualties, different forms of mobile hospitals should be adopted according to the needs to carry out a rescue at the disaster site.^3^

## DEVELOPMENT CHARACTERISTICS OF MOBILE HOSPITALS

At present, according to the differences of transport media, the mobile hospitals can be categorized into 3 types: terrestrial, floating, and flying. The terrestrial mobile hospital is the most common type, which includes tent hospital, vehicle hospital, shelter hospital, and so forth. The terrestrial mobile hospital is modular. The number of units, spatial organization, and clinical options could be altered as needed. From the point of view of spatial structure, the shelter hospital could respond immediately to disaster calls and immediate departure from the disaster zone when the situation worsens, and it must meet the requirements of flatness for supporting terrain. While the tent hospital has a large available space and low requirements for terrain, its disadvantages are long in deployment and withdrawal time. At present, the configuration forms of terrestrial mobile hospitals in various countries include mainly the shelter combination represented by the United States and France, the tent combination represented by Russia and Italy, and the shelter and tent combination represented by Germany and China.^3,4^

Shelter hospitals were first developed by developed countries, such as France, Germany, and the United States, which could be divided into non-expansion shelter and expansion shelter from the perspective of structure.^5^ The non-expansion shelter is basically transformed from a container, while the expansion shelter adopts a folding structure, the area of the structure before unfolding is the same as that of the non-expansion shelter, and the usable area after unfolding will expand. Typically, the types of expansion shelter structure can be divided into folding type, bellows type, and drawing type according to the way of retraction and unfolding.^3^ Although the structure of the shelter varies, in the face of some special rescue-site terrain, the medical shelter is not easy to deploy, so it cannot achieve a rapid and effective medical rescue.

The floating hospital is a self-contained, seagoing medical facility. As early as the end of the 1980s, Germany combined the medical shelter and cargo ship for reconstruction and realized the miniaturization of the hospital ship.^6^ The hospital ship named *Mercy* is currently used in the United States for the world’s disaster rescue and humanitarian operations.^7^ *Peace Ark*, which is the name of China’s hospital ship, has carried out dozens of humanitarian missions since its commission, treating over 180 000 patients around the world.^8^ Floating hospitals could only reach destinations near water and required a considerable amount of space to dock the ship and provide access for boats to transport the patients and staff.^1^

The flying hospital had been adopted by many of the world’s Air Forces,^9^ non-profit organizations, and governments. The flying hospital could provide surgical hospital services and acute medical care in disaster situations, or humanitarian relief.^10,11^ Moreover, aeromedical evacuation provided fast transportation of wounded persons at disaster sites to hospitals.^12^

## PRACTICES OF MOBILE HOSPITALS IN DISASTER RESPONSE

In this section, we collected the data from literature, websites, and scientific reports, to describe and discuss the disaster response of mobile hospitals in recent years.

**FIGURE 1:**
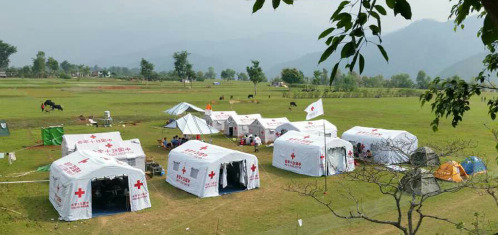
Tent Mobile Hospital Used by Chinese Medical Team in Nepal Earthquake Rescue.

As seen in Table 1, terrestrial mobile hospitals were most commonly used. In the earthquake in Haiti in 2010, the United States established the first tent hospital in Prince Lane of Haiti, which consists of the first 2 tent units for simple medical rescue, and transferred to the tent hospital with operation and intensive care functions composed of 4 tent units due to demand^20,21^; in 2013, the strong typhoon Haiyan swept across the central region of the Philippines, damaging infrastructure including hospitals. The medical teams sent by Israel and South Korea, under the command of Philippine authorities, assisted the damaged local hospitals and set up tent hospitals near the local hospitals^22,23^; according to the situation of the rescue site, China’s medical team set up a mobile hospital of inflatable tents in the mountainous area in the 2015 Nepal earthquake^24,25^ (Figure 1). Shelter hospitals played an important role in the rescue during 2 major earthquakes in China: Wenchuan and Yushu earthquakes.

**TABLE 1:** Disaster Response of Mobile Hospitals in Recent Years

Disaster Type	Date	Site	Country of Medical Team	Types of Mobile Hospitals
Flood	6-6-2001	Houston, America	America	Tent hospital^13^
Earthquake	12-26-2003	Iran	America	Tent hospital
Tsunami	11-26-2004	Indonesia	China, America	Tent hospital^12^
Earthquake	10-8-2005	Pakistan	China, America	Tent hospital^12^
Earthquake	5-12-2008	Wenchuan, China	China	Shelter hospital
Earthquake	1-12-2010	Haiti	America, China, France, Canada	Tent hospital^12, 14-16^
Earthquake	4-14-2010	Qinghai, China	China	Shelter hospital
Typhoon	11-8-2013	Philippines	Israel, South Korea	Tent hospital
			China	Hospital ship,^17^ tent hospital
Earthquake	4-25-2015	Nepal	China, Israel	Tent hospital^18,19^
Explosion*	8-12-2015	Tianjin, China	China	Vehicle hospital
COVID-19	12-2019	Wuhan, China	China	Vehicle hospital

*Notes:* *Explosion: At 22:51:46 on August 12, 2015, a fire and explosion occurred in the dangerous goods warehouse of Ruihai company located in Tianjin port, Binhai New Area, Tianjin. In this accident, the total explosion energy is about 450 tons of a TNT equivalent.

**FIGURE 2:**
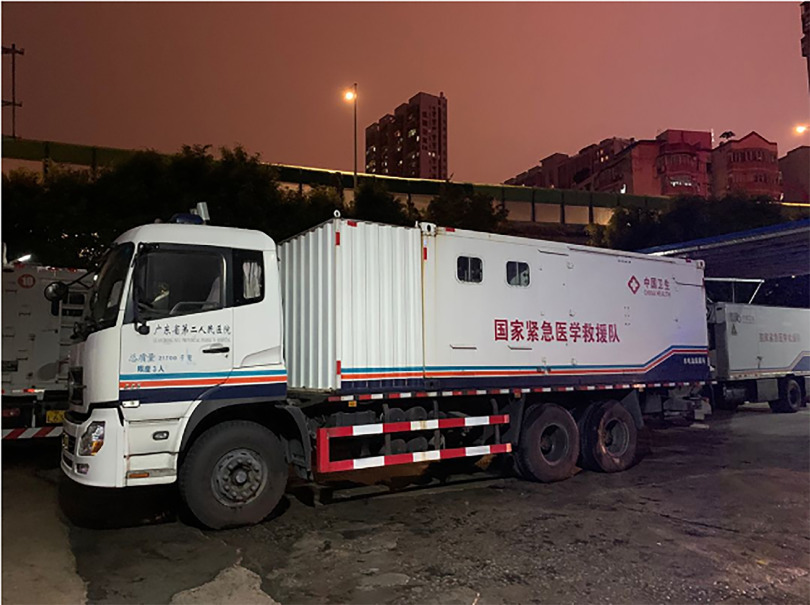
Rescue Material Delivery Vehicle of National Emergency Medical Rescue Team of the Second People's Hospital of Guangdong Province.

The vehicle hospital is also a main form of a terrestrial mobile hospital. After SARS in 2003, China’s disaster medical rescue system has been greatly developed. With the support of the central and local governments, the National Health Commission of China has established 4 categories (emergency medical rescue team, emergency infectious disease prevention and control team, emergency response team for poisoning, nuclear and radiation emergency health emergency team) and 49 national health emergency rescue teams. These teams are equipped with vehicle hospitals (Figure 2). This year’s outbreak of the novel coronavirus pneumonia named *coronavirus disease 2019 (COVID-19)* emerged in Wuhan, China. To deal with the surging demands of medical care and cut off the route of transmission to protect susceptible people, multiple national emergency rescue teams rushed to Wuhan through vehicle hospitals, and received and treated COVID-19 patients with mild symptoms. The stadiums were transformed into a temporary ward. A microorganism inspection vehicle and mobile CT vehicle were assembled to detect patients infected with COVID-19. The World Health Organization expert team highly praised this effective measure taken by China to deal with the epidemic.

The flying hospital is another kind of mobile hospital that can respond quickly to disaster. A flying hospital using a modified Lockheed L-1011 TriStar 50 jet aircraft landed outside of San Salvador, which supported medical assistance for 7000 patients, of which 187 included life-changing surgeries.^11^ Furthermore, air transport is the fastest mean of delivering the medical resource to the scene of disaster, or evacuation of wounded persons at disaster sites. In 2010, France proposed air transportation and delivery of medical equipment required by some mobile hospitals in the Haiti earthquake rescue.^26^ In the 2010 Haiti earthquake rescue, the Canadian medical team delivered mobile hospitals through air transportation.^27^ After a 6.5-magnitude earthquake hit Yunnan Province of China, the Il-76 transport plane of the Ministry of Emergency Situations (as the competent department of civil defense in Russia) took off from Ramenskoye Airport on the outskirts of Moscow, delivering humanitarian aid materials to the disaster area.^28^ In 2006, 35 firefighters were trapped during a forest fire in Greater Khingan Range, Heilongjiang province. The China International Search and Rescue team transferred these 35 severe casualties to Peking for treatment by using a modified airliner.^12^

A hospital ship refers to an unarmed service ship specially used for sea reception, treatment, and evacuation of the wounded and the dead. Continuing Promise 2011 was a 5-month humanitarian assistance/disaster response military aboard the hospital ship *Comfort*, which deployed to Central and South Americas and the Caribbean between April and September 2011^29^; In 2013, during the Philippines super typhoon disaster rescue, as the United Kingdom’s initial response to this disaster, HMS DARING was diverted from her deployment to take part in humanitarian aid, named Operation PATWIN.^30^ From November 24 to December 10, 2013, China’s hospital ship *Peace Ark* was deployed to the Leyte Gulf in the Philippines to provide humanitarian medical relief in Tacloban after Typhoon Haiyan.^31^

## LESSONS FROM THE FIELD

In the research process of a mobile hospital, innovation breakthroughs in various fields should be able to withstand the test of practice. Only in practice can new problems be found and solved. Among them, the valuable experience gained in the disaster rescue operation has played a crucial role. In a series of disaster rescues, there are still many aspects that need to be improved in the current application of the mobile hospital.

First, there is the problem of limited mobile transportation. After a disaster, it is very important to deliver the mobile hospital to the disaster site reasonably and effectively. At present, most of the terrestrial mobile hospitals rely on road and railway transportation. However, when the road or railway is damaged, the transportation time of the mobile hospital will be delayed, which may result in missing the golden time of rescue. During the Yushu earthquake in China, the locomotive transportation of a shelter hospital rushed to the disaster site overnight, driving 53 hours and 2350 kilometers to reach the disaster area^32^; like the Ya’an earthquake rescue in China, the shelter hospital did not give full play to the role of disaster rescue in the face of problems such as long distance and road damage.^6^

Second, special imaging and laboratory examination equipment should be considered, for example, adding a CT scan unit. In a disaster rescue, especially an earthquake, brain injury is the main problem of emergency treatment, and the operation of brain injury needs to use CT for accurate positioning.^33,34^ At the same time of adding necessary medical units, referring to the French practice, a modular unit combination is carried out according to needs, including devices, drugs, and equipment in each unit, forming several groups of combination formation of different functional types.^35^

Third, the internal equipment of the mobile hospital should be kept updated, especially the rescue medical equipment. For example, in the Nepal earthquake, it was proposed that due to the imperfection of the equipment, independent operation cannot be performed in the field hospital.^24^ At the same time, there is a growing need to formulate unified standards for mobile hospitals and form an orderly mechanism and system through the standards, so as to achieve standardized and unified management in the disaster site, especially during the international rescue operation.

## FUTURE DEVELOPMENT OF THE MOBILE HOSPITAL

### Integration and Application of Intelligent Medicine

The applications of machine learning (ML) and big data (BD) have been increasing rapidly in the mobile hospital, including medical imaging (MI), telemedicine, and computer-aided diagnosis (CAD) system. These technologies have become the mainstream of mobile hospitals and will be an important sign of future mobile hospitals.

In MI, the artificial neural network is the backbone of ML and deep learning.^36^ An important driver of the emergence of artificial intelligence (AI) in MI has been the enhancement of visual recognition using AI in radiology to produce lower error rates than the human observer.^37^ Specific capabilities of MI include detection and classification of lesions, automated image segmentation, extraction of radiomic features and study triage, and image reconstruction.^38-41^

The combination of a network and the medical field gave birth to telemedicine. The implementation of telemedicine can improve the uneven distribution of regional medical resources; reduce the working pressure of medical staff; shorten the distance between medical staff and patients and medical staff; and improve the timeliness of monitoring, diagnosis, and treatment. In surgical care,^42^ telemedicine technologies^43^ have been used to provide pre- and postoperative surgical consultations and monitoring, as well as surgical teleconferencing^44^ and education across borders.^45^ The continuous development of telemedicine relies on the continuous innovation of network communication technology; the latest fifth generation wireless systems (5G) is bringing significant changes to mobile communication and other related industries by virtue of its advantages of high data rate and low latency. To fight COVID-19, China Mobile, China Unicom, and China Telecom have recently applied 5G technology in high speed data transmission, telemedicine, and telemonitors in Huoshenshan Hospital in Wuhan.

**FIGURE 3:**
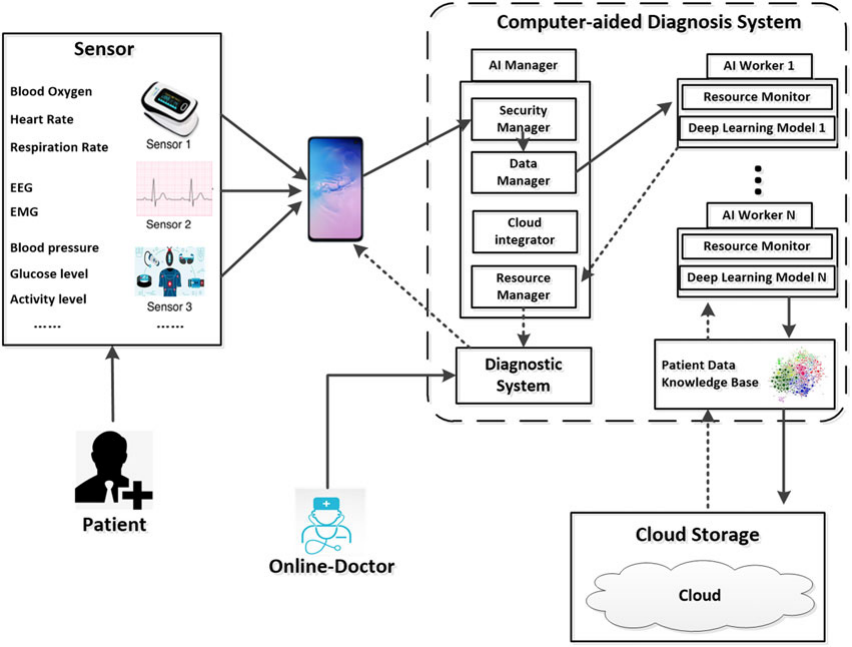
Computer-Aided Diagnosis System Architecture. (The solid lines indicate the input data and their direction, and the dashed lines indicate data-out and their direction.)

In 10 years, AI will create preliminary radiology reports about screenings for everything. The Alibaba Group has already developed an AI CT diagnosis technology to assist COVID-19 detection. The AI system could make acceptable diagnostic decisions within 20 seconds, with an accuracy rate of 96%.^46^ In the future, a computer will show us lesions right before our eyes,^47,48^ and be able to identify the 12 characteristics of cancer cells so that it will be able to diagnose it more accurately and faster than a pathologist can.^49^ Recently, IBM teamed up for therapy for diabetic patients by sharing 30 million data of patients with diabetes from clinical databases and 86 million data of patients with prediabetes. Mobile ultrasound, Google Glass, and other mobile devices have already been adopted in medical fields, and data from those wearable devices will be sent to a medical center where clinical review software will be used for the diagnosis and the treatment of patients.^50,51^ Telemedicine using a health spot center or prehospital care will become more prevalent. Figure 3 shows a CAD Architecture, comprising 3 parts: the sensor, CAD, and cloud storage. In the future, all of the new technologies and characteristic will be integrated into Mobile Hospital, telemedicine, computer-aided detection, and surgical robot.

### Rapid Deployment Capability

Rapid deployment of mobile hospitals in the disaster site is the most basic requirement for effective medical treatment. To achieve the rapid deployment capability, in addition to the aforementioned requirements of modular integration, it also requires the improvement of the nature of each unit and the level of mobile transportation. For example, the mobile emergency unit (MEU) developed in the United States is a kind of freight container, which can be used as an independent operating room, rehabilitation room, and patient ward under various medical equipment configurations. The combined medical equipment forms a hospital that can meet the needs of emergency medical resources in the disaster. Furthermore, the MEUs can be transported by helicopter.^52^ The mobile hospital’s own problems such as bulky materials, difficult structure development and collection, and limited space will cause serious impacts. The limitation of mobile transportation will probably lead to the disconnection of rescue equipment and personnel, which cannot reach the site at the same time. Therefore, the design and transportation of mobile hospitals is the focus of future research, so that mobile hospitals can better adapt to the needs of disaster rescue, so as to achieve the goal of rapid deployment capacity.

### Modularization and Standardization

In the face of future rescue, it can greatly improve the rescue efficiency by standardizing the unit design of the mobile hospital, grouping and classifying the modules according to the functions of each unit, taking targeted and effective choices in the face of different rescue situations, and combining the units. At the same time, it’s necessary to constantly update and improve the treatment system. In addition to the basic operation unit, inspection unit, emergency unit, ward unit, support unit, and technical support unit, it’s important to appropriately add specialized treatment units, including otolaryngology eye unit, obstetrics and gynecology unit, pediatrics unit, and infectious department unit, so as to improve the hospital scale standard that the whole can reach. In the 2015 Nepal earthquake, the mobile hospital jointly established by medical teams from China, Russia, and other countries in Durbar Square was subdivided and classified in the medical units, and different treatment units were added, which effectively improved the rescue efficiency and won the praise of the local people.^53^

## CONCLUSION

With the continuous development and innovation of science and technology, modern mobile hospitals will play an important role in the task of disaster response in the future. As the main force of emergency mobile medical support, mobile hospitals are bound to take on important medical support tasks. It can be predicted that the application of the BD system, AI-assisted diagnosis and treatment, and telemedicine based on new generation wireless communication technology will effectively improve the abilities of mobile hospitals.
